# High-Throughput Analysis from Complex Matrices: Acoustic Ejection Mass Spectrometry from Phase-Separated Fluid Samples

**DOI:** 10.3390/metabo11110789

**Published:** 2021-11-18

**Authors:** Yuzhu Guo, Michael Forbush, Thomas R. Covey, Lucien Ghislain, Chang Liu

**Affiliations:** 1 SCIEX, 71 Four Valley Drive, Concord, ON L4K 4V8, Canada; yuzhu.guo@sciex.com (Y.G.); tom.covey@sciex.com (T.R.C.); 2 Beckman Coulter Life Sciences, 170 Rose Orchard Way, San Jose, CA 95134, USA; mforbush@beckman.com (M.F.); lghislain@beckman.com (L.G.)

**Keywords:** acoustic ejection mass spectrometry, high-throughput analysis, sample preparation, liquid–liquid extraction

## Abstract

Acoustic ejection mass spectrometry is a novel high-throughput analytical technology that delivers high reproducibility without carryover observed. It eliminates the chromatography step used to separate analytes from matrix components. Fully-automated liquid–liquid extraction is widely used for sample cleanup, especially in high-throughput applications. We introduce a workflow for direct AEMS analysis from phase-separated liquid samples and explore high-throughput analysis from complex matrices. We demonstrate the quantitative determination of fentanyl from urine using this two-phase AEMS approach, with a LOD lower than 1 ng/mL, quantitation precision of 15%, and accuracy better than ±10% over the range of evaluation (1–100 ng/mL). This workflow offers simplified sample preparation and higher analytical throughput for some bioanalytical applications, in comparison to an LC-MS based approach.

## 1. Introduction

Mass spectrometry (MS) provides the high-sensitivity, high-fidelity, and high-specificity essential for various chemical and biological quantitation workflows, including drug discovery [1], forensic analysis [2], food safety [3], and environmental monitoring [4]. While MS has made tremendous strides, there remain challenges, including high-throughput sample introduction and time-consuming sample preparation. For aqueous samples, liquid chromatography (LC) is employed as the primary sample introduction method. Although proven effective, there is a throughput mismatch with MS that limits its practical use in high-throughput applications. Typical LC sample times are greater than 10 s, while MS sample times are measured in ms [5]. In addition, complex samples require a time and labor-intensive sample clean-up with LC-MS, to reduce ionization suppression and improve system robustness.

To address this analytical throughput bottleneck, various new MS ionization technologies have been developed that increase sampling rates to 1 s-per-sample or faster. These include matrix-assisted laser desorption/ionization MS (MALDI-MS) [6,7,8,9], desorption electrospray ionization MS (DESI-MS) [10], laser diode thermal desorption ionization MS (LDTD-MS), and acoustic mist ionization MS (AMI-MS) [11]. Although proven for some assays, the ionization suppression associated with direct MS injection approaches that omit the cleanup step remains a limitation for many complex assays. In electrospray ionization (ESI), the ionization suppression occurs when the dissolved solutes in the highly-charged droplets created during the electrospray process begin to limit the rate of ion production and introduce nonlinearities in the relationship between the number of analyte molecules and the response [12]. The high concentration of analyte and endogenous materials from biological and other sources, particularly those with surface active properties, are the major contributors. Careful optimization of the buffer composition and/or the sample cleanup prior to the analysis is required for some workflows [13].

Acoustic ejection mass spectrometry (AEMS) is a recently commercialized technology in which low nanoliter sample volumes are acoustically dispensed from a microplate into a continuous flow of carrier solvent in an open-port interface (OPI) for subsequent ESI-MS [14,15,16]. Analytical throughputs faster than 1 Hz have been reported, and the use of the conventional ESI provides broad compound coverage, from small molecules to intact proteins. Since the sample matrix is diluted ~1000 folds within the OPI prior to ionization, ionization suppression is significantly reduced and even completely eliminated for some workflows, such as direct analysis from various in vitro drug discovery assays, detergent samples, and plasma [16,17,18,19,20]. This unique online dilution feature greatly simplifies sample preparation and accelerates assay turnaround.

In a published work on inhibitors of diacylglycerol *O*-acyltransferase-2 (DGAT2), a transmembrane enzyme involved in the triglyceride production pathway, Song et al. found high signal variation in the case of direct sample ejection from an aqueous reaction buffer, due to the low solubility of the target analytes [21]. Wen et al. successfully integrated in-well liquid–liquid extraction (LLE) with AEMS technology by direct droplet ejection from phase-separated samples to analyze targets from an organic phase [22]. Less than 6% signal CV and a robust Z’ of 0.69 was reported. In this study, we demonstrate a two-phase AEMS workflow on a commercial AEMS platform and explore applications, with a focus on ionization suppression reduction in complex matrices. As a case study, high-throughput quantitation of fentanyl in urine was successfully demonstrated, with <1 ng/mL LOD, quantitation precision <15% CV, and accuracy 90–110% over the range of evaluation (1–100 ng/mL).

## 2. Results and Discussion

### 2.1. Two-Phase Acoustic Calibration

Dynamic fluid analysis (DFA), a proprietary real-time signal processing algorithm, automatically determines the optimal acoustic power for droplet ejection using a perturbation method [23]. At low acoustic power, a small fluid ‘mound’ forms at the fluid meniscus; DFA utilizes acoustic echoes to measure variation of the mound height with acoustic power. DFA signal processing is complex and time consuming; as such, the algorithm can be optimized for speed, or for a wide range of fluid properties, but cannot manage both a high speed and a wide range of fluid properties. Figure S1 is a plot of fluid properties for water, pentanol, and octanol, showing surface tension (dyne/cm) and viscosity (centiPoise, cP), two key properties that determine the acoustic power needed for optimal acoustic droplet ejection. The acoustic liquid handlers use calibrations defined by a limited range of fluid properties to achieve a desired droplet transfer speed (droplet ejection repetition rate). These calibrations include a CP (crystallography protein), designed for the widest possible range of fluid properties at low speed (30 Hz), and a SP (surfactant protein), designed for high-speed transfers (500 Hz) of aqueous solutions containing surfactants. In addition, we consider microplate well geometry, because the well forms a resonant cavity that generates surface capillary waves. The working range of well volumes is specific to the microplate form factor and composition. The two-phase fluid class was initially developed for a phase-separated system with a layer of 1-pentanol on top of an aqueous phase. The acoustic power must be increased for droplet ejection from higher viscosity fluids such as pentanol and octanol. The droplet ejection repetition rate was 30 Hz, or one droplet ejection every ~30 ms. Droplet ejection requires less than 1 ms, the balance of 29 ms allows time for the fluid meniscus to stabilize after droplet ejection.

**Figure 1:**
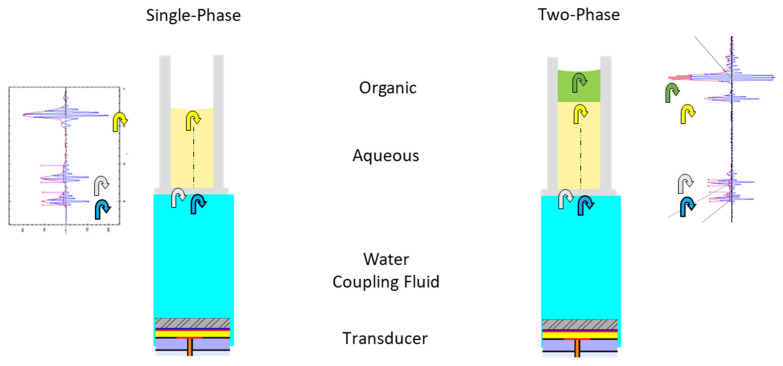
Schematic comparison of a single-phase and two-phase acoustic droplet ejection system.

Figure 1 is a schematic comparison of a single-phase and two-phase fluid system, showing the acoustic transducer, coupling fluid (Milli-Q H_2_O), aqueous phase, and organic phase in a microplate well. An acoustic excitation (ping) from the transducer travels through the coupling fluid into the microplate well, generating reflections (echoes) at each interface. Echo waveforms are shown adjacent to the diagrams aligned to the corresponding interface. There are four echoes in a two-phase system, the additional echo coming from the aqueous–organic fluid interface. We utilized a 3D phase doppler interferometric droplet detector (Artium Technologies, Inc., Sunnyvale, CA, USA) for real-time measurement of droplet diameter and velocity in three dimensions. Figure S2 shows the variation of droplet volume over a range of fluids for the CP and two-phase fluid classes. Ten droplets were transferred from 384 wells for each measurement. In the CP case, we made measurements of both low-fill (20 µL) and high fill (50 µL). Error bars represent the CV for 384 measurements. For CP, the droplet volume ranges from a low of 1.9 nL for 100% DMSO, to nearly 3.5 nL for water and 50% acetonitrile:H_2_O. In this study, this CP calibration was used for testing single-phase aqueous samples (data shown in Figure 3). For a two-phase system with aqueous/1-pentanol with droplet ejection from the top organic layer, the droplet volume was 1.4 nL.

**Figure 2:**
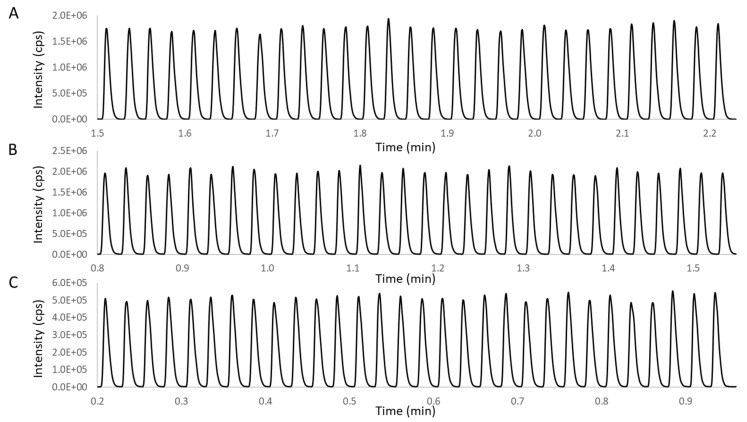
AEMS chronograms for the two-phase solution system of (**A**) water and 1-pentanol, (**B**) PBS and 1-pentanol, and (**C**) water containing 2% DMSO (*v/v*) and 1-pentanol.

The performance of the two-phase calibration was further evaluated on a commercial AEMS system (Echo^®^ MS), by monitoring ion counts from dextromethorphan dosed in various aqueous solvents, including water, PBS, and water containing 2% (*v*/*v*) DMSO. As shown in Figure 2, direct ejection from in-well LLE samples generated highly reproducible ion count signals (less than 5% peak area CV, N = 30), without any missed MS peaks. This demonstrated both the high volumetric precision of acoustically dispensed droplets and the well-controlled droplet placement for quantitative capture of the entire droplet within the OPI. We note that the signal level differs among the three solvent groups, according to analyte partitioning between 1-pentanol and the aqueous phase.

### 2.2. Two-Phase AEMS Performance

**Figure 3:**
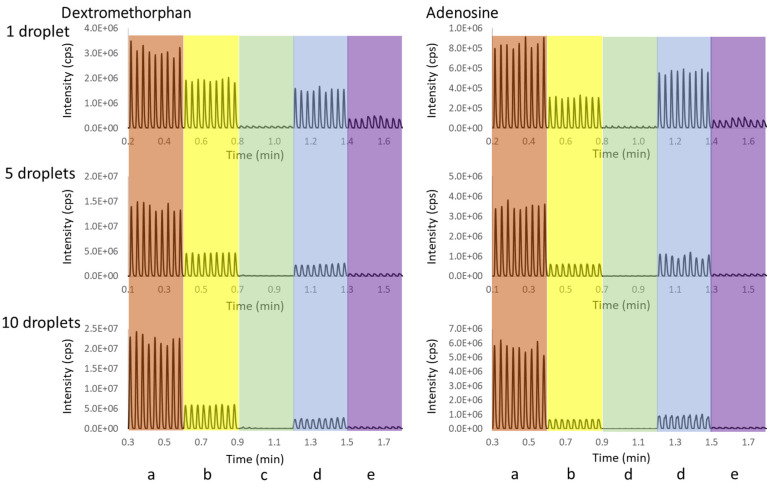
AEMS chronograms for the ejection of 1 µM dextromethorphan and adenosine in (**a**) water (red), (**b**) PBS (yellow), (**c**) water containing 1% PEG-400 (green), (**d**) water containing 200% CMC Triton X-100 (blue), and (**e**) urine (purple). The ejection volume was 1, 5, and 10 drops. There were nine ejections for each condition, which were triplicate ejections from the three sample wells.

We further evaluated a variety of in-well LLE conditions, including five different aqueous matrices (water, PBS, water containing 1% PEG-400, water containing 200% CMC Triton X-100, and urine) each containing two analytes with different hydrophobicities (dextromethorphan, octanol logP = 3.75, and adenosine, octanol logP = −1.05). To establish a baseline, single-phase aqueous samples were analyzed first, without the in-well LLE. Figure 3 shows the chronogram for these samples, with ejection volumes of 1, 5, and 10 droplets. Although highly reproducible data were observed within each group, a signal loss due to ionization suppression by the co-eluting sample matrix was observed for both analytes, despite the significant (>1000 folds) in-line dilution that occurred within the OPI [15]. The matrix effect of 1% PEG 400 and urine is more severe than PBS and 200% CMC Triton X-100. The signal from the urine matrix for both analytes was reduced to less than 5% of that from pure water. As expected, the reduction of analyte signal due to the ionization suppression was more severe with increased sample loading volume.

**Figure 4:**
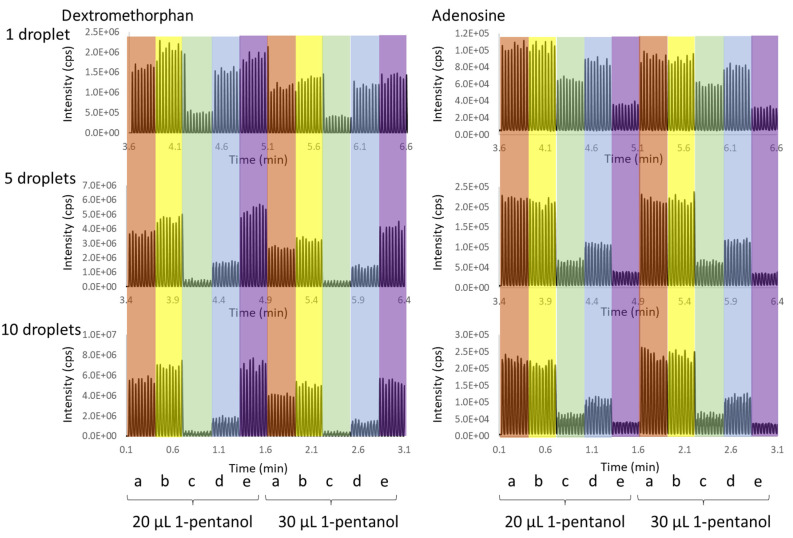
AEMS chronograms for the ejection from in-well LLE samples of 1-pentanol (20 and 30 uL) and various solutions (25 uL), containing dextromethorphan and adenosine in (**a**) water (red), (**b**) PBS (yellow), (**c**) water containing 1% PEG-400 (green), (**d**) water containing 200% CMC Triton X-100 (blue), and (**e**) urine (purple). The ejection volume was 1, 5, and 10 drops. There were nine ejections for each condition, which were the triplicate ejections from the three sample wells.

**Figure 5:**
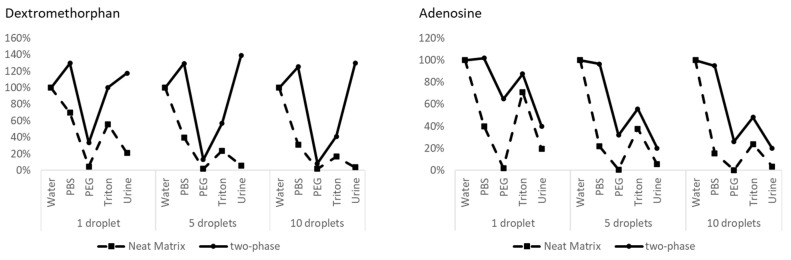
Signal level relative to the sample in water (neat matrix) from the different analyzed sample systems for both analytes.

The same sample set was then tested via in-well LLE, by adding two different volumes of 1-pentanol (20 and 30 µL). After the extraction, these two-phase samples were directly analyzed with the AEMS system using two-phase acoustic calibration; the chronograms are shown in Figure 4. The good data reproducibility within each group is evidence of a robust acoustic droplet ejection performance with the two-phase acoustic calibration. Samples with 30 µL 1-pentanol generated lower signals than the 20 µL group, due to the extra sample dilution. Comparing with the direct aqueous phase loading without the 1-pentanol extraction, in-well LLE significantly reduces ionization suppression from salts (e.g., PBS as the aqueous solution) for both analytes, even for a high sample loading volume (i.e., 10 droplets). Salts selectively partition to the aqueous phase, with a low partition level in the organic phase. For the hydrophobic species (dextromethorphan), a similar signal enhancement was observed from biological matrix (urine) across the sample ejection volume range: 1, 5, and 10 droplets. Ionization suppression was minimal for LLE processed Triton X-100 samples with single droplet ejection, though it became more obvious with increased sample ejection volume. Some reduction of matrix ion suppression for LLE processed PEG samples was also observed at a low sample ejection volume, for both the hydrophilic (adenosine) and hydrophobic (dextromethorphan) analytes (Figure 5).

This in-well LLE system offers a simple sample preparation workflow and enhanced the analytical sensitivity for hydrophobic species from high-salt and biological matrices. Together with the benefits of AEMS, including the high analysis speed, no observed carryover, high precision, and accuracy, this platform enables high-throughput analysis workflows, with an example described in the next section.

### 2.3. Case Study, Fentanyl Analysis from Urine

The illicit use of fentanyl has been a leading cause of drug overdose deaths [24]. Urine is a common matrix for drug testing, because the sample collection is noninvasive and the large sample volume availability [25]. However, an extensive sample cleanup is required prior to the LC-MS analysis, due to the complexity of this biological matrix [26]. Robust approaches to simplifying sample preparation and increasing the analytical throughput are desired. Recently, solid-phase microextraction (SPME) has been used to extract fentanyl from urine samples for either LC-MS or OPI-MS analysis [27,28], with a minimum required performance level (MRPL) of 2 ng/mL, as set by the World Anti-Doping Agency (WADA) [29]. Although SPME-OPI-MS improves throughput to 10–15 s per sample, even higher analytical throughput and simpler sample preparation procedures are needed to meet demand for high-throughput sampling [27].

**Figure 6:**
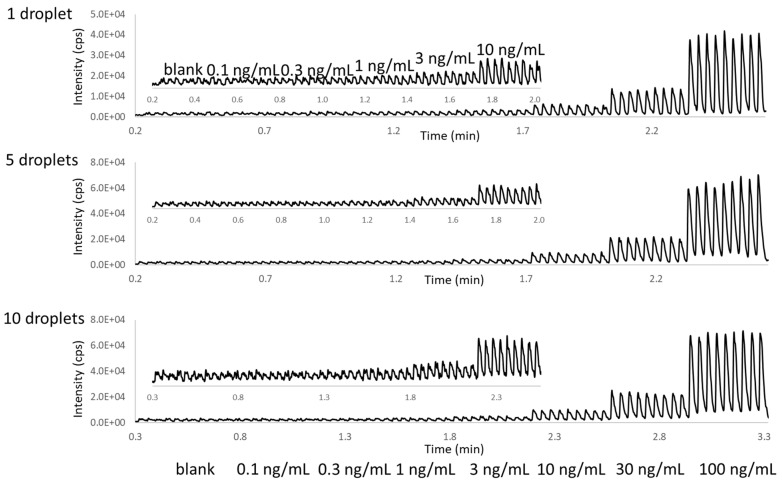
AEMS chronograms for the ejection from urine containing different concentrations of fentanyl (blank, 0.1, 0.3, 1, 3, 10, 30, and 100 ng/mL). There were nine ejections for each condition, which were triplicate ejections from the three sample wells. A magnified view of the first 6 concentrations (from blank to 10 ng/mL) is shown as an insert within each panel.

AEMS delivers an analytical throughput of seconds-per-sample along with high quantitation performance for direct ejection from complex matrices, including reaction buffers and plasma [16]. In this study, the analytical performance for direct ejection from neat urine was evaluated. As shown in Figure 6, although a good signal reproducibility was achieved, the sensitivity requirement could not be met, even with a 10-droplet ejection volume.

**Figure 7:**
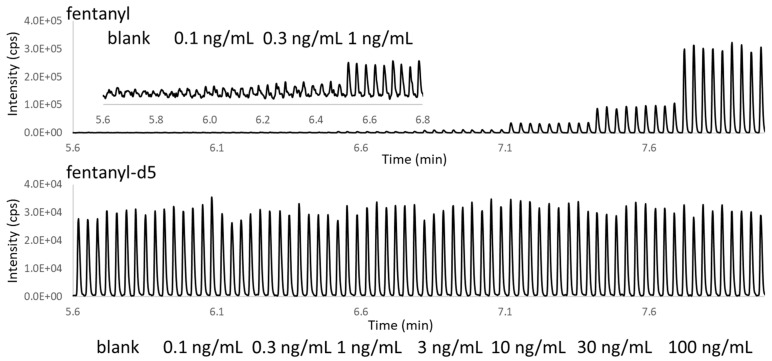
AEMS chronograms of fentanyl and the internal standard for the ejection from in-well LLE samples of 1-pentanol and urine containing different concentrations of fentanyl (blank, 0.1, 0.3, 1, 3, 10, 30, and 100 ng/mL). There were nine ejections for each condition, which were triplicate ejections from the three sample wells. The insert figure shows a zoomed-in view of the low-concentration range chronograms.

The analytical performance was then tested with the automation-friendly two-phase AEMS approach. As shown in Figure 7 and Table 1, AEMS analysis directly from in-well LLE with the two-phase acoustic calibration delivered a LOD better than 1 ng/mL, with just a single droplet ejection. This was due to the significantly reduced ionization suppression after LLE sample clean-up. Quantitation precision (<15% CV), accuracy (within ±10%) across all calibrants, and QC concentration points were observed with good linearity, from 1 to 100 ng/mL.

**Figure 8:**
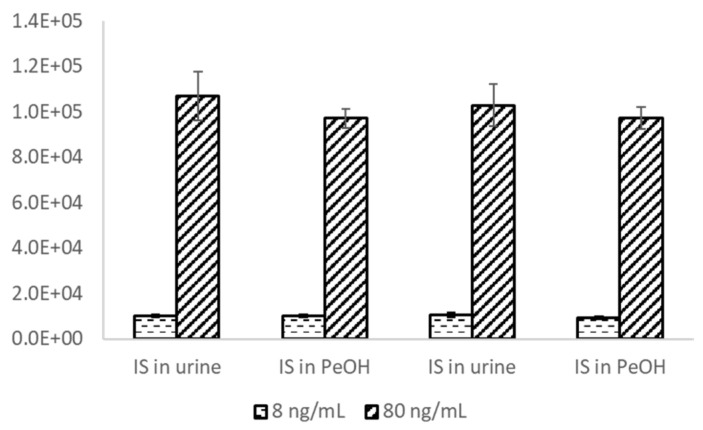
Signal intensity comparison of adding the internal standard in either the urine sample or the 1-pentanol. Each condition was repeated twice with two concentration levels tested.

To further demonstrate the simplicity of in-well LLE sample preparation, two different internal standard addition approaches were compared. Two concentration levels of fentanyl-D5 (8 and 80 ng/mL) were dosed, either into urine matrix or 1-pentanol extraction solvent, and the two-phase AEMS results were statistically the same as shown in Figure 8. This demonstrates an efficient LLE equilibrium and provides a further simplified procedure for sample preparation: pre-dosing the internal standard in 1-pentanol solvent, so that only a single liquid addition step is required for the sample plates. This feature also enables simpler standard addition quantitation workflows, where both the internal standard and analyte standard are dissolved in 1-pentanol and added to the sample as a signal step. This could be used for the analysis of complex samples where the blank matrix is challenging to acquire.

## 3. Materials and Methods

### 3.1. Reagents and Materials

Adenosine, phosphate buffered saline (PBS), polyethylene glycol (PEG) 400, Triton™ X-100, dimethyl sulfoxide (DMSO), and 1-pentanol were purchased from Sigma Aldrich (Oakville, ON, Canada). Dextromethorphan, fentanyl, and fentanyl-D5 were acquired from Cerilliant (Round Rock, TX, USA). Urine was purchased from BioIVT (Westbury, NY, USA). The HPLC-grade methanol and acetonitrile were bought from Caledon Laboratory Chemicals (Georgetown, ON, Canada) and J.T. Baker (Radnor, PA, USA), respectively. Deionized water (18 MΩ) was produced in-house using a Millipore Integral 10 water purification system (Billerica, MA, USA).

### 3.2. Sample Prepration

Samples without the in-well liquid–liquid extraction (LLE) were directly loaded into the microplate wells. Samples requiring in-well LLE contained 25 µL of aqueous samples and 20 µL 1-pentanol. The sample plate was shaken at 1000 RPM on an orbital shaker for 5 min, followed by centrifugation at 2000 RPM for 10 min.

### 3.3. AEMS System

A detailed description of the AEMS system has been provided elsewhere [12]. For this study, we installed research-grade CP and two-phase acoustic calibrations on the Echo^®^ MS system (SCIEX, Framingham, MA, USA). A SCIEX Triple Quad 6500+ was used as the detector and all data were collected in multiple reaction monitoring (MRM) mode (Q1: 272.2 to Q3: 215.1 for dextrometrhorphan, Q1: 268.1 to Q3: 136.1 for adenosine, Q1: 337.2 to Q3: 188.3 for fentanyl, and Q1: 342.2 to Q3: 188.3 for fentanl-D5). Carrier flow (methanol with 0.1% fomic acid) was optimized at 500 µL/min. NECO Diagnosis software was used to control the acoustic ejection, and the mass spectrometer was operated with SCIEX Analyst 1.7 software. The data were processed with a prototype version of MultiQuant software.

## 4. Conclusions

Direct analysis from in-well liquid–liquid extraction samples is demonstrated on the commercial AEMS platform. This simple and automation-friendly sample preparation approach mitigates ionization suppression, to enhance the analytical sensitivity for some bioanalytical assays with complex matrices. A stable dispensing performance was described for various aqueous phase compositions, including fentanyl in urine. This approach can be broadly applied for high-throughput analysis environments, when the target analyte hydrophilicity is different from the sample matrix, and to improve assay performance and reduce MS system contamination.

## Figures and Tables

**Table 1:** Analytical performance using an AEMS system, analyzing the in well LLE samples for fentanyl quantitation from urine.

	Concentration (ng/mL)	CV (%)	Accuracy (%)
	0	-	-
	1	13.4	98.8
Calibrants	3	9.7	94.4
	10	6.6	101.6
	30	9.0	100.0
	100	8.9	100.0
	2	8.5	106.8
QC	15	5.3	106.3
	75	4.6	108.3

## Data Availability

Raw data are available upon request from the corresponding author.

## Supplementary Materials

The following are available online at https://www.mdpi.com/article/10.3390/metabo11110789/s1, Figure S1: Fluid properties of water, pentanol and octanol. Figure S2: The measured volume of ten acoustically ejected droplets from different solvent systems.
