# Supplementary material for: High-Throughput Analysis from Complex Matrices: Acoustic Ejection Mass Spectrometry from Phase-Separated Fluid Samples
Source: Metabolites. 2021 Nov 18;11(11):789. doi: 10.3390/metabo11110789 (PMC8618436; doi:10.3390/metabo11110789)
Supplement: Supplementary file 1 [file metabolites-11-00789-s001.zip › metabolites-1463943-supplementary.pdf]

Supporting Information

High-Throughput Analysis from Complex Matrices: Acoustic Ejection Mass Spectrometry from Phase-Separated Fluid Samples

Yuzhu Guo [1], Michael Forbush [2], Thomas R. Covey [1], Lucien Ghislain [2] and Chang Liu [1,*]

[1] SCIEX, 71 Four Valley Drive, Concord, ON L4K 4V8, Canada; yuzhu.guo@sciex.com (Y.G.); tom.covey@sciex.com (T.R.C.)
[2] Beckman Coulter Life Sciences, 170 Rose Orchard Way, San Jose, CA 95134, USA; mforbush@beckman.com (M.F.); lghislain@beckman.com (L.G.)
* Correspondence: chang.liu@sciex.com

This file includes:

- Supplemental Figures S1 and S2

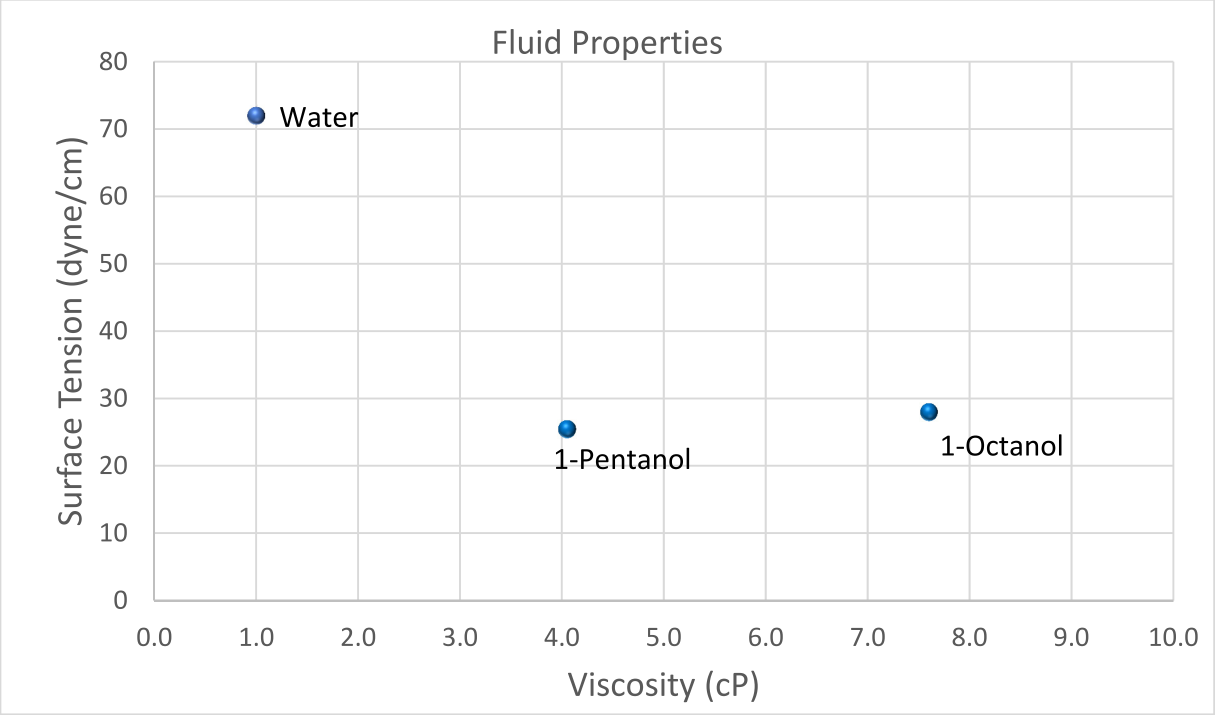

Figure S1. Fluid properties of water, 1-pentanol and 1-octanol.

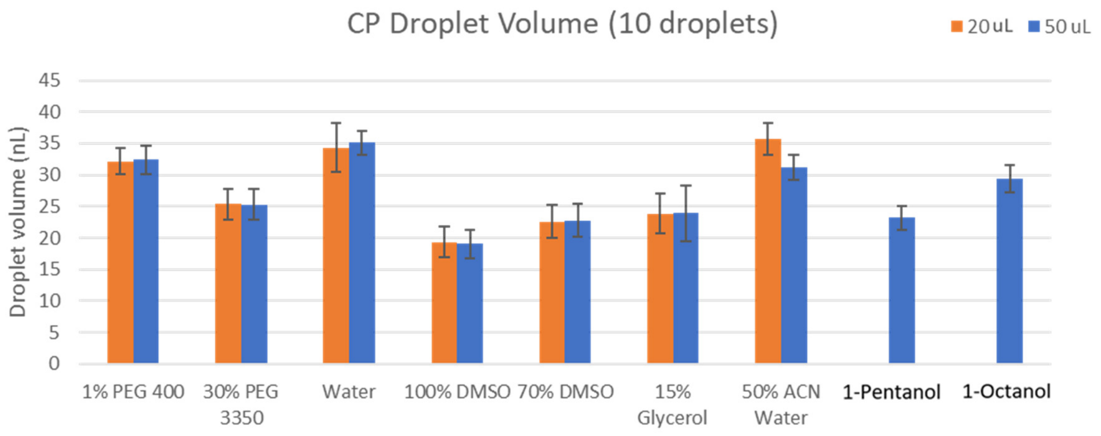

Figure S2. The measured volume of ten acoustically ejected droplets from different solvent systems.
